# From awareness to action: investigating the impact of big-five teamwork model awareness on rationing of nursing care and patient-centered care

**DOI:** 10.1186/s12912-025-02711-y

**Published:** 2025-02-04

**Authors:** Heba Mohamed Al-Anwer Ali Ashour, Ebtsam Aly Omer Abou Hashish, Nadia Hassan Ali Awad

**Affiliations:** Department of Nursing Administration, Faculty of Nursing, Alexandria University, Alexandria, Egypt

**Keywords:** Big five teamwork, Rationing of nursing care, Nurses, Missed nursing care, Patient-centered care

## Abstract

**Background:**

Nurses are the cornerstone of healthcare delivery, playing a pivotal role in ensuring safe, high-quality, and patient-centered care. However, the significant demands on their time and resources often lead to the rationing or omission of essential nursing care activities, undermining patient outcomes. As frontline caregivers, nurses’ ability to collaborate effectively within teams is critical to overcoming these challenges. The Big Five Teamwork Model, which emphasizes trust, communication, and leadership, offers a transformative approach to strengthening teamwork and addressing barriers to comprehensive nursing care.

**Aim:**

This study aimed to assess the impact of the Big Five Teamwork Awareness Sessions (Big 5TWAS) on the rationing of nursing care and patient-centered care.

**Methods:**

A quasi-experimental pre-post-test design was conducted in critical care units at an Egyptian university hospital. A convenience sample of 60 nurses and 31 admitted patients participated in the study. Big 5TWAS interventions were implemented for nurses, and study variables were measured using nursing teamwork surveys, the Rationing of Nursing Care Observational Checklist (RONCO), and patient-centered care questionnaires before and after the intervention. Data were analyzed using descriptive and inferential statistics.

**Results:**

The Big 5 Teamwork Awareness Sessions (Big 5TWAS) demonstrated a significant impact on the study variables. Post-sessions, there was a significant decrease in rationing of nursing care scores and significant increases in nursing teamwork and patient-centered care scores (*p* ≤ 0.001). In addition to the significant correlation values, regression analysis revealed that overall teamwork accounted for approximately 22% of the variance in rationing of nursing care (R² = 0.224, *p* = 0.015), with trust as the significant predictor. Furthermore, teamwork explained approximately 80% of the variance in patient-centered care (R² = 0.801, *p* < 0.001), with trust, backup, and team leadership emerging as significant predictors.

**Conclusion and recommendations:**

The study underscores the critical role of teamwork in reducing the rationing of nursing care and enhancing patient-centered care in critical care units. The Big 5TWAS was effective in fostering improved team dynamics, trust, and leadership, which translated into better care delivery. These findings highlight the need for ongoing inter-professional training and teamwork-enhancing strategies led by nurse managers to sustain and amplify these improvements. By focusing on teamwork, healthcare institutions can ensure high-quality patient outcomes and minimize missed nursing care practices.

## Introduction

Nurses are recognized as a core part of healthcare, making up the largest portion of the healthcare workforce. Numerous studies have highlighted the essential role of nursing in delivering high-quality care [1, 2]. Nurses focus on patient-centered care, aiming to meet patient needs to enhance the quality and safety of healthcare delivery [3]. Despite their critical role, nurses often face significant demands on their time and resources, which can lead to the omission or incomplete performance of necessary nursing activities, a phenomenon known as implicit rationing of care [4]. In this context, identifying strategies to alleviate the challenges faced by nurses is crucial. The Big-Five Teamwork Model (Big5TWAS), which emphasizes teamwork and collaboration, presents a promising approach to addressing these issues [5]. By fostering teamwork, this model has the potential to reduce the rationing of care and improve patient-centered care. This study aims to examine the impact of Big-Five Teamwork Model awareness sessions on the rationing of nursing care and patient-centered care, providing insights into how enhancing teamwork can lead to improved healthcare outcomes.

## Background

### Big five teamwork

**Fig. 1:**
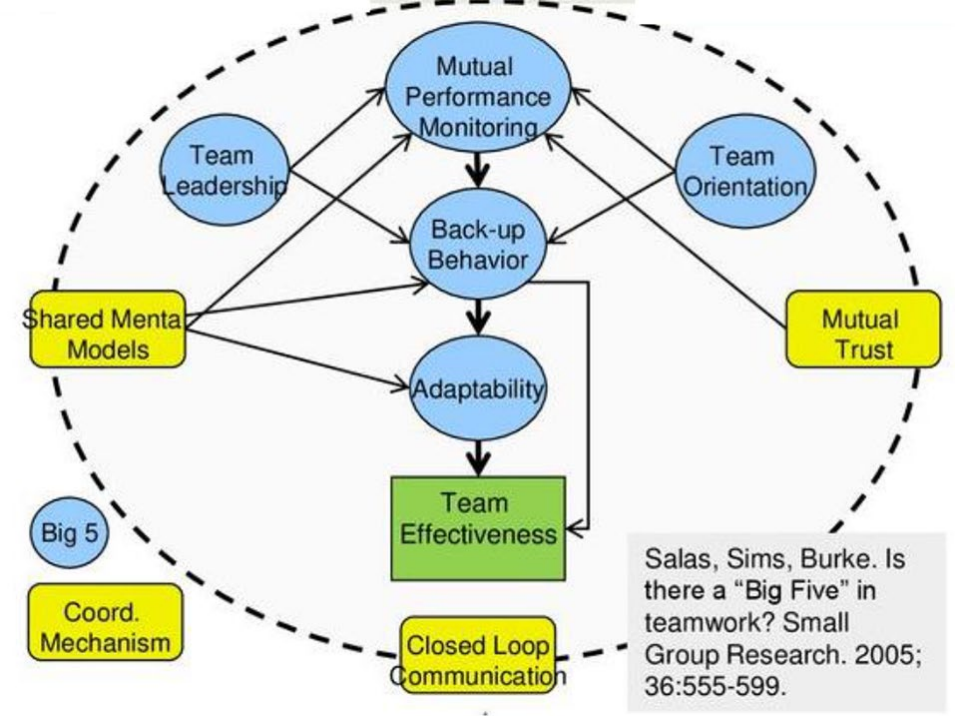
Big-five teamwork model (adapted from Salas et al.) [7]

Effective teamwork is crucial in healthcare environments for ensuring efficient and safe patient care. Research demonstrates that strong teamwork can decrease medical and nursing errors, leading to increased patient satisfaction and improved healthcare results [6]. This study utilizes Salas et al.‘s model [7] which outlines the Big Five components as essential elements of effective teamwork. According to this model, teamwork involves a blend of each team member’s thoughts, actions, and emotions, supporting coordinated and adaptable performance to achieve collective goals that enhance overall outcomes [7]. See Fig. 1.

The Big Five Teamwork Model highlights five essential components along with three coordinating mechanisms. These key components include team leadership, mutual performance monitoring, backup behavior, adaptability, and team orientation. Additionally, these elements rely on coordinating functions—such as shared mental models, closed-loop communication, and mutual trust—which are essential for effective teamwork and vary in significance based on the team’s stage and specific tasks. Team leadership provides guidance, structure, and support, either from a formal leader or through contributions from other team members. Team orientation emphasizes prioritizing team success over individual accomplishments. Mutual performance monitoring involves team members maintaining awareness of each other’s performance. Backup behavior includes assisting teammates when necessary, while adaptability refers to adjusting strategies in response to changing conditions [7].

The three coordinating mechanisms in effective teamwork include shared mental models, closed-loop communication, and mutual trust. Shared mental models facilitate a common understanding among team members regarding tasks, roles, and workflows. Closed-loop communication ensures active information sharing, with acknowledgment and verification to confirm message receipt. Mutual trust involves confidence in team members’ dedication to collective goals and the team’s best interests [7, 8]. Effective teamwork is thus widely recognized as a critical approach to minimize care rationing and foster patient-centered, efficient care delivery [6].

### Rationing of nursing care (RONC)

In healthcare, rationing refers to managing limited resources to address patient needs effectively and achieve optimal outcomes [9]. Rationing of Nursing Care (RONC) involves deliberately delaying or withholding essential nursing interventions necessary for patients’ health and well-being. This practice can result in negative outcomes, such as medication errors, patient falls, pressure ulcers, and other complications [10]. In healthcare literature, rationing is generally divided into explicit and implicit types. Explicit rationing is the formal distribution of limited resources, guided by legal standards, specific criteria, and institutional policies. Implicit rationing, on the other hand, relies on healthcare providers’ professional judgment and situational factors, rather than defined regulatory frameworks, to allocate resources in real-time clinical settings [11, 12].

This study examines implicit rationing in nursing, where limitations such as inadequate staffing, time pressures, insufficient supplies, and skill mismatches interfere with the completion of necessary nursing duties. This issue, often referred to as “unfinished nursing care,” “nursing care left undone,” or “missed nursing care” [13, 14], has implications beyond specific tasks, affecting overall patient outcomes and nursing workload management. It involves prioritizing critical aspects of patient care, including thorough health assessments, ongoing safety monitoring, essential nursing interventions, accurate documentation, emotional support, rehabilitation, patient education, and privacy protection [15–17]. Implicit rationing illustrates the delicate balance between available resources and quality care, highlighting the urgent need for resource management and policy measures to alleviate its adverse effects on healthcare delivery [9].

#### Rationing of nursing care and the big five team model

Several factors, including team dynamics as outlined in the Big Five Team Model, influence the rationing of nursing care, significantly influencing how nurses and healthcare teams coordinate resources and manage patient care [7]. Studies indicate that effective team dynamics, as described by the Big Five Team Model, can reduce the frequency of implicit rationing in nursing. For example, strong team leadership promotes clear communication and structured decision-making, which helps to clarify resource allocation and prioritize care tasks [15]. Mutual performance monitoring enhances accountability among team members, facilitating prompt intervention and task completion, which reduces instances of missed nursing care [16]. Therefore, understanding these dynamics is essential for promoting patient safety and quality care. Applying the principles of the Big Five Team Model can help decrease implicit rationing and improve overall healthcare outcomes and patient satisfaction. Encouraging a culture of transparency and open communication can also support the reporting and management of rationing-related incidents, thereby enhancing care quality and safety [10].

Numerous studies highlight concerns regarding the adverse effects of rationed nursing care on patient outcomes, linking it to increased mortality rates, more frequent falls, higher occurrences of hospital-acquired pressure ulcers, and a greater prevalence of hospital-acquired infections. This issue jeopardizes both patient safety and care quality, especially in teaching and university hospitals facing resource limitations [9–11, 18, 19]. Evidence from both national and international research points to widespread care rationing across various healthcare environments. For example, Bragadóttir et al. [2] found that approximately 54% of nurses consistently rationed at least one care task, while Diab and Ebrahim (2019) identified rationing rates ranging between 30% and 70%, depending on the healthcare facility [20]. These findings underscore the pressing need for strategies to mitigate rationing and enhance patient safety. Recent research suggests that interventions focused on bolstering teamwork can significantly reduce rationed care and improve patient outcomes [21, 22].

### Patient-centered care (PCC)

Patient-centered care (PCC) is fundamental to delivering high-quality healthcare, shaped by both organizational structures and care processes. It is linked to enhanced patient satisfaction, clinical outcomes, and economic benefits [23]. PCC has gained considerable focus in healthcare, highlighting its role in improving patient experiences [10]. Though definitions may vary, PCC generally involves recognizing each patient as an individual and providing supportive, compassionate, and empathetic nursing care. This approach represents a shift from traditional, provider-centered, disease-oriented models to systems that prioritize the preferences, needs, and experiences of patients [24]. Key elements of PCC include compassion, skill, communication, respect, and comfort. Compassion requires nurses to empathize with patients’ perspectives and offer emotional support. Skill refers to delivering evidence-based care efficiently, especially in critical situations. Communication emphasizes clear, effective interactions among healthcare providers to advocate for patient needs. Respect values patients as individuals and involves families in care decisions. Comfort, essential for overall well-being, addresses both the physical and emotional needs of patients [24].

The core principles of patient-centered care (PCC) align well with the behaviors described in the Big Five Team Model. Compassion and respect reflect empathy and advocacy, while expertise ensures the safe and effective delivery of care. Additionally, effective communication aligns with the model’s emphasis on mutual performance monitoring and coordination among team members [7, 24]. Combining PCC with the Big Five Team Model promotes a comprehensive approach to healthcare, enhancing both patient satisfaction and clinical outcomes. Conversely, studies show that implicit rationing of nursing care has a negative relationship with patient-centered care. Specifically, lower levels of rationed care are associated with greater patient understanding, more thorough information provision, and stronger recognition of personalized treatment needs [23]. Therefore, to reinforce patient-centered care, it is crucial to improve the nursing work environment and minimize implicit care rationing.

### Study framework

**Fig. 2:**
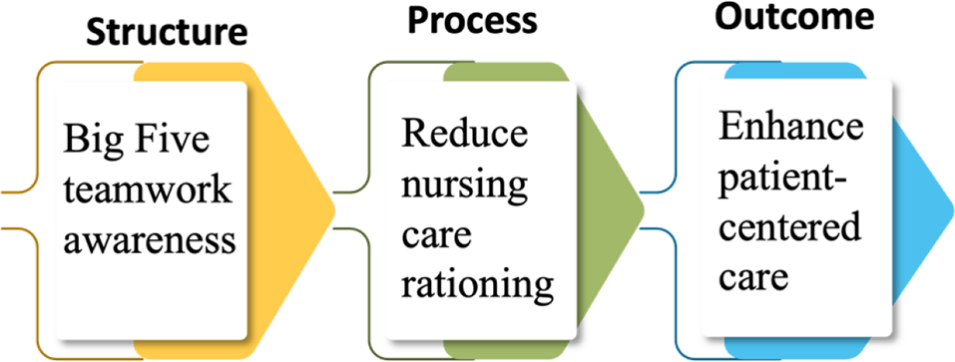
Researchers’ proposed conceptual framework

This study is structured around Donabedian’s quality model, which focuses on the relationship between healthcare structure, care processes, and outcomes. According to Donabedian’s model, a strong structural foundation is essential for promoting effective care processes, which in turn lead to better patient outcomes [25]. In this study’s framework, the researchers propose that the Big Five Teamwork Model (the structural factor) can reduce nursing care rationing (the process variable) and enhance patient-centered care (the outcome variable). This framework is supported by research demonstrating that training in Big Five teamwork principles is essential for reducing nursing care rationing and effectively addressing patient needs [23]. This approach underscores the importance of strengthening healthcare team structures through the application of Big Five teamwork principles to improve care processes and ultimately enhance patient-centered care outcomes (see Fig. 2).

### Research hypothesis

#### H1

The Big Five teamwork awareness sessions will significantly improve nursing teamwork.

#### H2

The Big Five teamwork awareness sessions will significantly enhance patient-centered care.

#### H3

The Big Five teamwork awareness sessions will significantly reduce nursing care rationing.

### Significance of study

Nurses working in healthcare settings, particularly in teaching and university hospitals, face substantial challenges that hinder their ability to deliver optimal care. These include limited resources, heavy workloads, high levels of stress, and chronic staffing shortages, which collectively place immense pressure on nursing professionals [19, 26]. Such demands often impose the rationing of nursing care, where essential nursing tasks are omitted or delayed due to insufficient time or resources. This phenomenon significantly compromises care quality and adversely impacts patient safety and outcomes [19, 26–28].

Patient-centered care (PCC), recognized as a cornerstone of quality healthcare, emphasizes prioritizing patients’ preferences, needs, and values in clinical decisions [24]. However, achieving consistent PCC remains challenging due to pervasive care rationing in healthcare systems.This rationing, frequently driven by resource constraints, excessive workloads, and inadequate staffing, limits nurses’ ability to engage fully with patients and provide holistic, individualized care [9, 16, 19, 20].

Research highlights that care rationing jeopardizes patient safety, satisfaction, and health outcomes by increasing adverse events and undermining therapeutic nurse-patient relationships decisions. Despite its importance in healthcare quality frameworks, PCC implementation is often fragmented, with systemic barriers impeding its consistent delivery decisions [10]. Moreover, although the growing emphasis on patient involvement in care decisions, many individuals still feel excluded, particularly during complex choices.

Addressing these barriers is essential to enhance care delivery and foster a supportive healthcare environment for both patients and providers [21, 23]. Nursing research plays a crucial role in bridging these gaps by promoting patient-centered care models that address patients’ emotional, cultural, social, and physical well-being.

This study aims to bridge this critical gap by investigating the Big Five Teamwork Model as an intervention to enhance teamwork, reduce care rationing, and promote PCC. The study’s findings might provide insights into actionable strategies to empower nurses, optimize teamwork, and develop organizational policies prioritizing patient-centered approaches while mitigating care rationing. These findings could emphasize the importance of structured teamwork training programs in advancing healthcare quality and improving patient outcomes.

### Aim of the study

This study aimed to investigate the impact of Big Five nursing teamwork awareness sessions (Big5TWAS) on the rationing of nursing care and patient-centered care.

## Method

### Study design, setting, and participants

The study employed a quasi-experimental design, specifically a one-group pretest-posttest approach, within the critical care units of Alexandria Main University Hospital in Alexandria, Egypt.

The study included two participant groups. The first group comprised staff nurses working in critical care units. A convenient sample of 60 nurses was selected from a total of 263, based on the inclusion criterion of having at least six months of experience in a critical care environment. Sample size calculation was performed using G Power software to ensure statistical reliability, targeting a power of 0.9, an alpha level of 0.05, and an effect size of 0.5, resulting in a minimum required sample size of 60 nurses. This group was central to assessing the effects of the interventions on nursing care practices.

The second group consisted of patients admitted to these critical care units in the prior three months. From a total of 124 admitted patients, 31 (representing 25%) were included in the study. These patients met specific criteria: they were alert, willing to participate, hospitalized for over 24 h, and cared for by nurses who had participated in the study’s awareness sessions.

### Study instruments

Five tools were utilized to evaluate different aspects before and after the team intervention awareness session:

#### Demographic characteristics form

Designed by the researchers, this form collected demographic information from staff nurses, including details such as age, gender, educational background, years of experience, prior participation in training courses, and nurse-to-patient ratio.

#### Nursing teamwork survey (NTS)

Created by Kalisch et al. [29], this survey evaluates nurses’ perceptions of the five core characteristics of teamwork. It includes 33 items divided into five categories: trust (7 items), team orientation (9 items), backup (6 items), shared mental model (7 items), and team leadership (4 items). Each item is rated on a 5-point Likert scale, from (1) “never” to (5) “always,” with total scores ranging from 33 to 165. Higher scores indicate stronger teamwork among nurses. The tool demonstrates high reliability, with a Cronbach’s alpha of 0.79 for the total scale.

#### Rationing of nursing care observational checklist (RONCOC)

Developed by the researchers through an extensive literature review [14–17], this checklist assesses the extent of implicit rationing in nursing care. It includes 60 items organized into six categories: nursing interventions (21 items), assessment, monitoring, and safety (18 items), documentation (5 items), emotional support (3 items), rehabilitation, instruction, and education (5 items), and patient privacy and nursing ethics (8 items). Responses are scored on a four-point scale (not applicable = 0, complete = 1, incomplete = 2, not done = 3), based on observed instances of incomplete nursing tasks. Total scores range from 0 to 180, with higher scores reflecting greater levels of care rationing.

To ensure reliability, the tool underwent inter-rater reliability testing during a pilot phase. Observers were trained in the consistent application of the checklist, and inter-rater reliability was assessed using the Intraclass Correlation Coefficient (ICC). The ICC value of 0.85 indicates a high level of agreement among the raters. The tool also demonstrated strong internal reliability, with a Cronbach’s alpha of 0.78 for the full scale.

#### Patient-centered critical care nursing questionnaire (PCCNP)

Created by Hong and Kang [24], this questionnaire has two sections. The first section gathers patient socio-demographic information, including age, sex, marital status, education level, and length of hospital stay. The second section features 20 items across five dimensions—compassion, expertise, communication, respect, and comfort—with each dimension containing four items. Responses are recorded on a 4-point Likert scale, from (1) “strongly disagree” to (4) “strongly agree,” with total scores ranging from 20 to 80. Higher scores indicate greater levels of patient-centered care. The tool has demonstrated strong reliability, with a Cronbach’s alpha of 0.84 for the overall scale.

#### Participant program evaluation form

Designed by the researchers to evaluate nurses’ perceptions of the program’s effectiveness, this form includes 16 items across four dimensions: content (5 items), environment (3 items), presenter effectiveness (4 items), and instructional methods (4 items). Responses are recorded on a 4-point Likert scale, ranging from (1) “strongly disagree” to (4) “strongly agree.” Total scores range from 16 to 64, with higher scores indicating greater perceived program effectiveness.

### Validity and reliability

All study questionnaires, except the RONCOC, were translated into Arabic and rigorously assessed for translation accuracy, content validity, and cultural relevance by a panel of five academic experts. After translation, the tools were back-translated into English by language specialists. The researcher and panel reviewed these back-translations to ensure precision and minimize validity risks. The Content Validity Index (CVI) achieved a perfect score of 1.00 for all instruments, reflecting outstanding content validity. Reliability tests confirmed the reliability of each tool, with Cronbach’s alpha coefficients exceeding 0.70. Additionally, a pilot study involving 10% of the total sample was conducted, leading to minor adjustments to enhance clarity and facilitate smooth data collection for the main study.

### Data collection and ethical consideration

The study protocol was reviewed and approved by the Research Ethics Committee of the Faculty of Nursing at the University of Alexandria (IRB-00013620). Formal permission to carry out the research and gather data was secured from the hospital following a detailed explanation of the study objectives. All participants provided written informed consent after being fully informed about the study’s purpose. For observational aspects of the study, witnessed consent was obtained from first-line nurse managers within the units. Confidentiality and participant anonymity were strictly upheld throughout the research. Additionally, participants were assured of their voluntary participation and informed of their right to withdraw from the study at any time without any repercussions.

### Phases of data collection

**Fig. 3:**
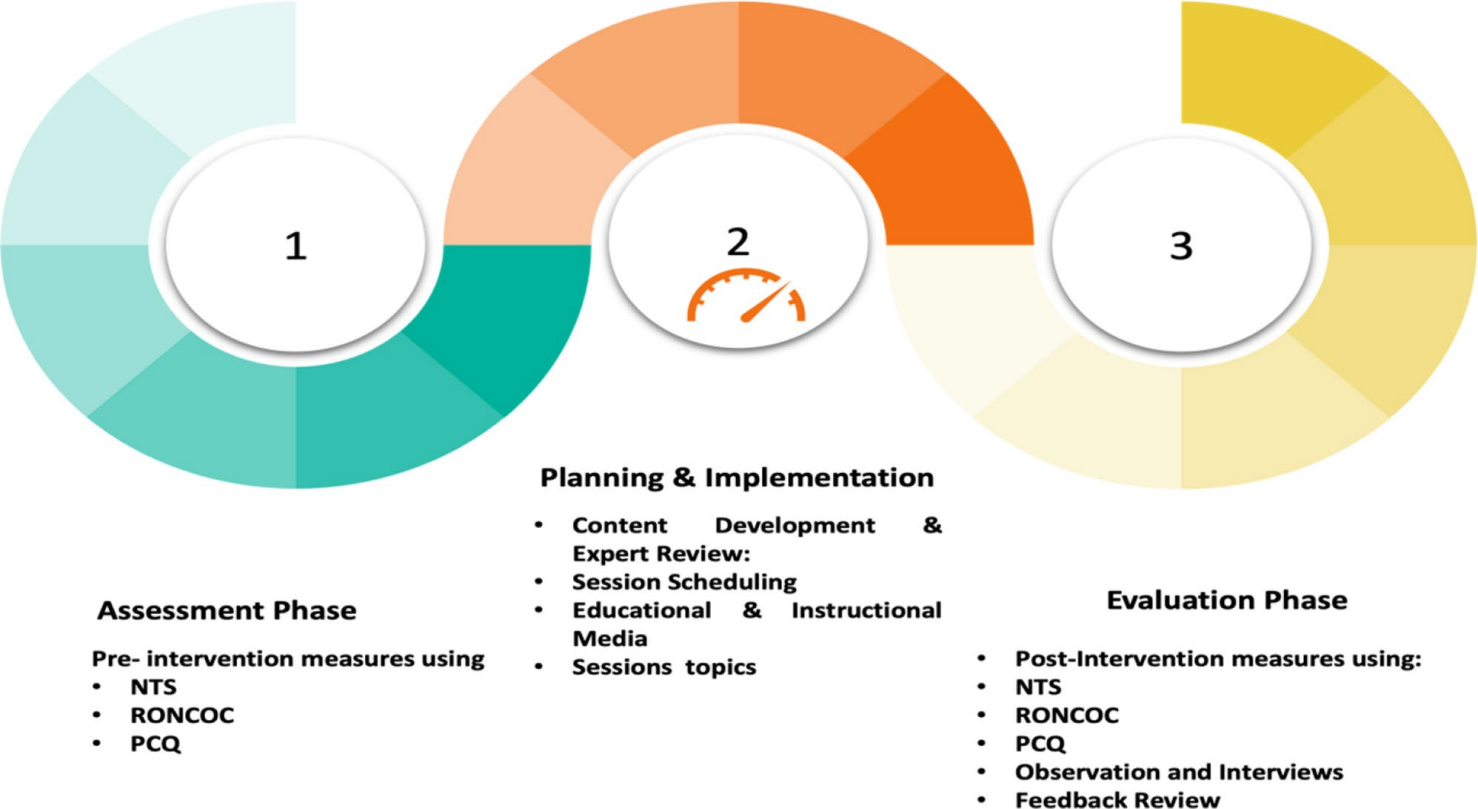
Phases of data collection

The data were collected through the following phases (see Fig. 3):

#### Assessment phase

In the initial assessment phase, three primary instruments were employed to establish baseline data. The Nursing Teamwork Survey (NTS) was distributed to staff nurses as a self-administered questionnaire to evaluate their perceptions of teamwork across different dimensions. Concurrently, the Rationing of Nursing Care Observational Checklist (RONCOC) was implemented through systematic, continuous observations conducted by the researchers. To minimize the Hawthorne effect, observers were already familiar to the nurses, reducing potential behavior changes. Additionally, nurses were informed about the study’s general purpose without revealing specific tasks under evaluation. Observations were conducted discreetly during morning, evening, and night shifts, with each nurse observed separately, resulting in a total of 180 observations for the 60 nurses. The researchers documented the status of nursing tasks, categorizing them as completed, incomplete, delayed, or not performed. Additionally, the Patient-Centered Care Questionnaire (PCQ) involved structured interviews with individual patients. These interviews, lasting around 30 min per session, were conducted daily with 5–6 patients, following an explanation of the study’s purpose.

#### The planning and implementation phases

In the planning and implementation phase of the Big Five Teamwork Sessions (Big 5TWSs), the session content was developed through a literature review and insights from the initial assessment phase. A detailed handout summarizing session topics was reviewed and refined by a panel of five experts from Nursing Administration and Nursing Education to ensure clarity and relevance. Sessions were scheduled with input from participants to fit their availability and workloads and were held in hospital conference rooms. The content included an overview of teamwork principles, effective teamwork practices using the “Big Five Framework,” the five core components, and the three coordinating mechanisms. Each session was structured with clear objectives, relevant topics, diverse educational methods, and an evaluation method. An introductory orientation outlining the training goals preceded each session, followed by a Q&A segment to address staff nurses’ questions and feedback. Educational strategies included lectures, group discussions, role-playing, video presentations, brainstorming, and instructional media like handouts and PowerPoint slides.

#### Evaluation phase

The evaluation phase commenced directly after implementing the Big Five Teamwork Sessions (Big 5 TWSs). Data collection in this phase used the same tools as the initial assessment: the self-administered Nursing Teamwork Survey (NTS) to evaluate nurses’ perceptions of teamwork, the Patient-Centered Critical Care Nursing Questionnaire (PCCNP) to assess patients’ views on patient-centered care, and the RONC observational checklist to monitor nursing care practices. Observations with the RONCOC tool followed the same structured format as the pre-intervention stage. Additionally, the researcher utilized the Participants’ Program Evaluation Questionnaire post-session to assess the perceived effectiveness of the Big 5 TWSs from the participants’ perspective. This data collection phase lasted four months, from April to July 2022, after which statistical analyses were conducted to interpret the findings.

### Data analysis

Data were analyzed using IBM SPSS software version 22. Descriptive statistics, including frequencies, percentages, means, and standard deviations, were used to summarize demographic data and study variables. The Kolmogorov-Smirnov test was applied to assess data normality. For normally distributed quantitative variables, ANOVA with repeated measures was used to evaluate changes across time points. For non-normally distributed paired data, the Wilcoxon signed-rank test (Z) was applied to compare pre- and post-intervention scores. Pearson’s correlation analysis explored relationships among study variables. Linear regression analysis was conducted to examine the predictive role of teamwork on nursing care rationing and patient-centered care. Cronbach’s alpha was used to assess the reliability of the study instruments. Statistical significance was set at ≤ 0.05 for all analyses, ensuring the rigor and reliability of the results.

## Results

### Participants’ characteristics

The demographic breakdown of the participating nurses indicated that most were female, representing 75.0% (*n* = 45). The largest age group was between 30 and 40 years, making up 43.3% (*n* = 26). In terms of marital status, 46.7% (*n* = 28) were married, and an equal percentage held a bachelor’s degree in nursing science. More than half of the nurses, 58.4% (*n* = 35), had over 10 years of professional experience, while 95.0% (*n* = 57) had not participated in any teamwork-related training courses. Regarding additional work hours, 51.7% (*n* = 31) worked 1–2 extra shifts weekly. In terms of patient assignments, the largest groups (41.7%) (*n* = 25) were each responsible for either one or two patients. Refer to Table 1 for more details.

For the patient demographic, the majority (58.0%) were aged 50–69 years. Males slightly outnumbered females at 54.8% (*n* = 17). Most patients (58.1%, *n* = 18) were married, and just over half, 51.6% (*n* = 16), stayed in the hospital for 3–7 days. Additionally, 54.8% (*n* = 17) of patients had received education. See Table 2 for further details.

**Table 1:** Distribution of study nurses according to demographic data (*N* = 60)

Demographic data	No.	%
**Sex**		
Male	15	25.0
Female	45	75.0
**Age (years)**		
Less than 30years	9	15.0
30–40	26	43.3
41–50	19	31.7
More than 50	6	10.0
**Educational preparation**		
Secondary nursing diploma	13	21.6
Technical health institute	19	31.7
Bachelor’s degree of nursing science	28	46.7
**Years of experience**		
Less than 5 years	8	13.3
5–10 years	17	28.3
More than 10 years	35	58.4
**Marital status**		
Single	22	33.3
Married	28	46.7
Divorced	12	20.0
**Attendance of teamwork training course**		
Not attended	57	95.0
Attended	3	5.0
**Extra time (overtime hours/week)**		
No extra hours	23	38.3
1–2 shift	31	51.7
3–4 shift	6	10.0
**Nurse to patient ratio**		
1–2 patients	25	41.7
3 patients	21	35.0
4 patients	14	23.3

**Table 2:** Distribution of study patients according to demographic data (*N* = 31)

Demographic data	No.	%
**Age**		
Less than 49 years	7	22.6
50–59 years	9	29.0
60–69 years	9	29.0
70 years and more	6	19.4
**Sex**		
Male	17	54.8
Female	14	45.2
**Marital status**		
Un married	13	41.9
Married	18	58.1
**Length of stay**		
3 days	4	12.9
3–7 days	16	51.6
More than 8 days	11	35.5
**Education**		
Literate	17	54.8
Illiterate	14	45.2

### The observed differences in the perceived teamwork, rationing of nursing care, and patient-centered care variables pre- and post-awareness sessions

Table 3 presents highly significant statistical differences in the mean percent scores of overall Big Five teamwork and its dimensions before and after Big 5 TWSs with a p-value of 0.001. The mean percent score for teamwork was 36.07 ± 3.69 before the sessions, compared to 63.96 ± 3.69 after the awareness sessions. All dimensions of teamwork showed highly significant differences before and after the awareness sessions (*p* ≤ 0.001), with the most notable increases observed in team leadership, backup, and shared mental model.

The table also reveals highly significant statistical differences in the mean percent scores of overall rationing of nursing care and its dimensions before and after the awareness sessions, with a p-value of 0.001. The mean percent score was 84.13 ± 3.55 before the sessions compared to 12.77 ± 3.63 after the sessions. The dimensions of rationing of nursing care most affected before the awareness sessions were nursing documentation, rehabilitation, nursing assessment, and nursing interventions, which showed significant improvement after the awareness sessions (*p* ≤ 0.001).

**Table 3:** The observed differences in the perceived teamwork and rationing of nursing care among nurses pre- and post-awareness sessions

Variables	Pre	Post	Z	*P*	
	Mean % ± SD.	Mean % ± SD.			
**Overall Nurses’ Teamwork**	36.07 ± 3.69	63.96 ± 3.69	6.736*	< 0.001^*^	
Trust	25.36 ± 8.98	78.81 ± 9.37	6.740*	< 0.001^*^	
Team Orientation	13.15 ± 5.31	69.31 ± 8.94	6.737*	< 0.001^*^	
Backup	19.38 ± 11.17	85.28 ± 4.50	6.741*	< 0.001^*^	
Shared Mental Model	25.77 ± 7.15	83.81 ± 4.29	6.717*	< 0.001^*^	
Team Leadership	23.13 ± 14.15	85.63 ± 10.18	6.757*	< 0.001^*^	
**Overall RONC**	84.13 ± 3.55	12.77 ± 3.63	6.738*	< 0.001^*^	
Nursing interventions	83.77 ± 9.89	14.26 ± 5.47	6.740*	< 0.001*	
Nursing assessment	91.27 ± 5.36	9.31 ± 7.53	6.765*	< 0.001*	
Nursing documentation	95.33 ± 9.99	9.67 ± 11.93	6.772*	< 0.001*	
Emotional support	73.89 ± 19.50	32.22 ± 13.68	5.515*	< 0.001*	
Rehabilitation	91.33 ± 12.33	11.33 ± 13.29	6.687*	< 0.001*	
Respecting patients’ privacy	64.33 ± 13.44	11.85 ± 12.19	6.735*	< 0.001*	

Z: Wilcoxon signed ranks test *: Statistically significant at *p* ≤ 0.05

Furthermore, Table 4 illustrates that there were highly statistically significant differences between the mean percent scores of patient-centered care and its dimensions before and after awareness sessions, with a p-value of < 0.001. The mean percent score was 32.47 ± 4.57 before the session, compared to 81.08 ± 3.98 after the awareness implementation. All dimensions showed highly significant differences before and after the awareness sessions (*p* ≤ 0.001), with the most notable increases observed in compassion, communication, and respect.

**Table 4:** The observed differences in the perceived patient centered care among patients pre- and post-awareness sessions

Patient centered care (PCC)	Pre	Post	Z	*P*
	Mean % ± SD.	Mean% ± SD.		
Compassion	34.68 ± 19.73	86.02 ± 8.44	4.870*	< 0.001^*^
Expertise	36.83 ± 19.81	77.69 ± 9.23	4.772*	< 0.001^*^
Communication	33.06 ± 14.51	80.91 ± 12.94	4.877*	< 0.001^*^
Comfort	22.31 ± 11.46	79.03 ± 8.30	4.876*	< 0.001^*^
Respect	35.48 ± 15.95	81.72 ± 12.06	4.869*	< 0.001^*^
**Overall PCC**	32.47 ± 4.57	81.08 ± 3.98	4.689*	< 0.001^*^

Z: Wilcoxon signed ranks test *: Statistically significant at *p* ≤ 0.05

Table 5 shows that the awareness sessions significantly impact the studied variables, revealing statistically significant differences in nurses’ rationing of nursing care, teamwork, and patient-centered care before and after the sessions, with a p-value of less than 0.001.

**Table 5:** Effect size for awareness sessions on overall rationing of nursing care, teamwork and patient centered care

Variables	Pre Mean% ± SD.	Post Mean% ± SD.	F	*P*	Effect size	Level
**Overall RONC**	109.5 ± 2.99	67.20 ± 2.41	3953.663	< 0.001^*^	0.985	Large Effect
Mean difference		**42.27 ± 3.67**				
**Overall Nurses’ teamwork**	80.62 ± 4.87	117.43 ± 4.87	1058.995	< 0.001^*^	0.947	Large Effect
Mean difference		**36.82 ± 6.81**				
**Overall PCC**	39.48 ± 2.74	68.65 ± 2.39	1072.247	< 0.001^*^	0.973	Large Effect
Mean difference		**29.16 ± 0.58**				

F: F test (ANOVA) with repeated measures *: Statistically significant at *p* ≤ 0.05

No Effect (< 0.2), Small Effect (0.2–<0.5), Intermediate Effect (0.5–<0.8), and Large Effect (More than 0.8)

### Correlation and regression analysis between teamwork and each of rationing of nursing care and patient-centered care after the awareness sessions

In addition to the correlation among the studied variables, the regression analysis demonstrated that nurses’ teamwork plays a significant predictive role in improving patient-centered care and reducing the rationing of nursing care. Overall, teamwork explained approximately 80% of the positive variance in patient-centered care (R² = 0.801, adjusted R² = 0.762, F = 20.171, *p* < 0.001), emphasizing its critical role in enhancing patient care experiences. Additionally, teamwork accounted for 22% of the negative variance in rationing of nursing care (R² = 0.224, adjusted R² = 0.153, F = 3.125, *p* = 0.015), highlighting its potential to reduce care rationing. Among the dimensions of teamwork, trust, backup, and team leadership were significant predictors of patient-centered care, while trust was the sole significant predictor in reducing rationing of nursing care. See Table 6.

**Table 6:** Correlation and regression analysis between teamwork and each of rationing of nursing care and patient-centered care after the awareness sessions

Teamwork	Rationing of nursing care^r1^					Patient-centered care^r2^				
	B	t	*p*	95% CI		B	t	*p*	95% CI	
				LL	UL				LL	UL
Trust	-0.058	-2.510	0.015*	-0.104	-0.012	0.561	4.337	< 0.001*	0.295	0.827
Team Orientation	-0.016	-0.719	0.475	-0.059	0.028	0.092	0.792	0.436	-0.332	0.147
Backup	-0.049	-0.913	0.365	-0.157	0.059	0.958	2.801	0.010*	0.254	1.663
Shared Mental Model	-0.034	-0.731	0.468	-0.129	0.060	0.100	0.453	0.654	-0.356	0.557
Team Leadership	-0.016	-0.705	0.484	-0.061	0.029	1.028	6.808	< 0.001*	0.717	1.339
**Overall**	R^2^ = 0.224, adjusted R^2^ = 0.153, F = 3.125, *p* = 0.015*					R^2^ = 0.801, adjusted R^2^ = 0.762, F = 20.171, *p* < 0.001*				

r^1^and r^2^ Pearson coefficient values with overall teamwork *r*=-0.316, *r* = 0.425, R^2^: Coefficient of determination, B: Unstandardized Coefficients t: t-test of significance, F, p: f and p values for the model, LL: Lower limit, UL: Upper Limit, CI: Confidence interval, *: statistically significant at *p* ≤ 0.05

### Nurses’ perception of awareness sessions’ effectiveness

Table 7 shows nurses’ perception of the awareness sessions’ effectiveness was evaluated immediately after completion. The overall program effectiveness scored highly, with a mean percent score of 90.24 ± 3.55. The presentation section received the highest rating at 93.06 ± 8.14, followed by instruction at 92.22 ± 6.49. Content and setting were also rated positively, with mean scores of 87.78 ± 8.78 and 87.96 ± 10.88, respectively. These results indicate that the nurses perceived the awareness sessions as highly effective.

**Table 7:** Nurses’ perception to awareness sessions’ effectiveness (*N* = 60)

Program effectiveness section	Mean % score
Content	87.78 ± 8.78
Setting	87.96 ± 10.88
Presentation	93.06 ± 8.14
Instruction	92.22 ± 6.49
Overall Program effectiveness	90.24 ± 3.55

## Discussion

The critical role of nurses in delivering safe, individualized, and holistic care is fundamental to healthcare quality [5]. In order to support these essential nursing functions, effective strategies are required to empower nurses to prioritize patient care and foster patient-centered practices across various healthcare settings. This study addresses this need by exploring the impact of Big 5 Teamwork Awareness on nursing care rationing and the enhancement of patient-centered care, highlighting a potentially transformative approach for improving healthcare outcomes.

The findings revealed notable improvements in nurses’ perceptions of teamwork and a reduction in the rationing of nursing care following the implementation of the Big 5 Teamwork Awareness Sessions (Big 5TWAS). Prior to the Big 5TWAS, nurses encountered substantial difficulties in delivering nursing care, particularly in high-demand environments like critical and intensive care units. Despite the essential role of teamwork, results indicated that nurses initially perceived teamwork as inadequate, with low levels reported across dimensions such as trust, backup, shared mental models, and team leadership. This may be attributed to a general lack of awareness about the significance of teamwork, as nurses reported limited exposure to training or workshops on this subject. Additionally, high levels of nursing care rationing were evident in areas such as documentation, rehabilitation, assessment, interventions, and providing emotional support, likely due to the intensive care requirements of patients in these units. This aligns with Griffiths et al.‘s systematic review, which highlights frequent missed care areas, including patient ambulation, new admission assessments, care planning, oral hygiene, interdisciplinary conferences, and documentation [30].

Numerous studies [31, 32] highlight the influence of factors such as staffing shortages, high patient admissions, disruptions to routine workflows, and the need to manage auxiliary services on challenges related to inadequate teamwork and the rationing of nursing care. Research by Hammad et al. [28] and Arslan et al. [33] further emphasizes how high patient loads and perceived staffing shortages contribute to missed care, though their findings show some variation. These elements have a direct effect on the quality of patient care, often compelling nurses to prioritize certain tasks and resort to rationing care—an important indicator of gaps in healthcare quality.

After implementing the Big 5TWAS, there was a marked rise in the mean scores for overall teamwork, reflecting a meaningful improvement in collaborative practices, including team leadership, backup support, and shared mental models. This enhancement is significant, as strong teamwork is essential for coordinated patient care and helps reduce the implicit rationing of nursing services. Additionally, the overall rationing of nursing care saw a notable decline, with a significant drop in the mean percentage score. Specific areas such as nursing documentation and rehabilitation also experienced considerable improvements following the sessions. These outcomes indicate that the sessions effectively addressed the root causes of care rationing and increased awareness, allowing nurses to better collaborate, allocate resources more strategically, and prioritize comprehensive patient care [16, 19].

These findings are consistent with prior research underscoring the role of teamwork, collaboration, and a supportive work environment in minimizing the rationing of nursing care. Zhao et al. [21] found that teamwork positively influences the reduction of implicit care rationing and enhances various aspects of nursing practice. The advantages include improved knowledge and skills, better communication, strengthened trust among team members, reduced turnover intentions, fair staffing levels, clear division of responsibilities, and a stronger sense of team unity. The study concluded that effective teamwork can substantially reduce implicit care rationing. Similarly, Bragadóttir et al. [2] noted that teamwork is vital for patient safety, emphasizing that strong teamwork is a critical component of a positive nursing work environment [2]. Evripidou et al. [34] also identified teamwork as an area with significant potential for developing strategies to address the challenges of rationing in nursing care (RONC).

After completing the Big 5TWAS, patients reported a noticeable improvement in compassionate and respectful interactions with nurses. This shift may be attributed to the sessions’ emphasis on the importance of compassionate, patient-centered care, which was also evident in nurses’ reflections during the final debriefing. Additionally, patients observed an increase in patient-centered care following the nurses’ participation in the awareness sessions, potentially reflecting higher patient satisfaction. This finding aligns with Costello et al. [6], who noted that effective teamwork can reduce nursing errors and improve patient outcomes, and Durand and Fleury [35], who showed that strong team collaboration positively impacts patient-centered perceptions.

The findings revealed a substantial effect size on teamwork, nursing care rationing, and patient-centered care following the implementation of the Big 5 Teamwork Awareness Sessions (Big 5TWAS). Regression analysis demonstrated that teamwork plays a predictive role in both reducing nursing care rationing and enhancing patient-centered care. The negative regression coefficient for teamwork and care rationing highlights that improved teamwork is a significant predictor of reduced rationing practices. Similarly, the positive regression coefficient for teamwork and patient-centered care indicates that teamwork strongly predicts better patient-centered care delivery. The strong predictive effects, along with the observed correlations, emphasize the critical role of teamwork in improving care quality and efficiency. These results support the study hypotheses 1–3 and align with previous studies, which have consistently demonstrated the close relationship between teamwork, care quality, and patient outcomes in healthcare settings, underscoring the value of teamwork-focused interventions in enhancing care processes and patient experiences [3, 36].

Prior studies have shown that the Big 5 Teamwork Training Program significantly improved nurses’ knowledge, skills, and attitudes toward teamwork while reducing missed nursing care instances [22, 37]. Similarly, Marguet and Ogaz [38] observed a decrease in missed care scores after the intervention. Bragadóttir et al. [2] and Nobahar et al. [39] also reported that nurses in intensive care units with strong teamwork experienced fewer cases of missed care. Together, these findings indicate that teamwork training programs like the Big 5TWAS can play a crucial role in enhancing nursing practices by minimizing care rationing and promoting patient-centered care, though results may vary depending on the setting and specific study conditions.

These findings are reinforced by nurses’ highly positive feedback on the Big 5TWAS, with high ratings across elements such as content, setting, presentation, and instructional resources. Many nurses shared qualitative reflections on the value of these sessions. For instance, one nurse expressed, “*The sessions covered all the critical teamwork aspects that we often overlook due to our busy schedules*.” Another noted, “*Participating in the Big 5TWAS has boosted my confidence in collaborating with my colleagues*, *which has greatly enhanced the quality of care I deliver to my patients*.”

Nurses also highlighted the role of the sessions in fostering more respectful, compassionate, and patient-centered care. One nurse shared, “*The training made me realize the importance of showing compassion and respect in every patient interaction. It’s not just about medical care but also about emotional support.”* Another nurse commented, “*The sessions taught us how to be more patient-centered*, *and I’ve noticed a big difference in how my patients respond to me now—they feel heard and cared for.”* The findings of this study align with previous research emphasizing the significant impact of structured awareness sessions and training programs on improving workplace variables among nurses. These initiatives have demonstrated transformative potential in fostering professional competencies, strengthening teamwork, and addressing challenges in healthcare delivery [40–42].

### Strengths and limitations

The study’s limitations should be considered when interpreting the results. The convenience sampling method from a single hospital setting, without the inclusion of a control group, restricts the broader applicability of the findings, as they may not reflect other settings or the wider nursing workforce. Additionally, the lack of random assignment for the intervention could introduce biases, as non-randomized selection may impact the study’s internal validity. While the Big Five Teamwork training showed potential in reducing nursing care rationing and enhancing patient-centered care, the lack of previous Big Five collaboration programs in this hospital might mean the outcomes partially reflect the novelty of the intervention. To strengthen validity and generalizability, future studies should utilize randomized control groups across diverse locations.

## Conclusion

In conclusion, this study highlights the transformative potential of the Big 5 Teamwork Awareness Sessions in nursing practice. The sessions led to significant improvements in teamwork perceptions, reduced care rationing, and fostered a patient-centered culture, all of which are essential in delivering high-quality healthcare. These findings underscore the importance of structured teamwork training as a strategic intervention for addressing challenges in nursing environments, such as resource limitations and high workloads, that can compromise care quality. The positive impact observed on both nursing practice and patient outcomes suggests that implementing teamwork-focused programs like Big 5TWAS could be a valuable investment in healthcare settings, enhancing nurse engagement and patient satisfaction alike. Future studies are encouraged to replicate this approach across diverse settings with controlled methodologies to strengthen the generalizability of these promising outcomes.

### Implications of the study

The current study brings forth several implications for nursing practice, management, and future research:

#### Implications for nursing practice and management

Hospital management should implement ongoing training and educational programs that emphasize inter-professional collaboration to enhance patient experience and effective teamwork. Establishing continuous monitoring systems for rationed nursing care and daily workload assessments is essential to ensuring proper staffing and the safety of care. Nurse managers should foster an environment that promotes effective teamwork and collaboration. To develop a teamwork-oriented environment, nurse managers should prioritize fostering effective collaboration among all healthcare disciplines. This can be achieved through regular multidisciplinary rounds, team meetings, and informal huddles, allowing each team member to contribute their expertise to patient care planning. Nurse managers should also ensure a balanced nurse-to-patient ratio by regularly assessing patient acuity and aligning it with the skill level and experience of nursing staff, using evidence-based staffing tools to meet care complexity demands. Additionally, nurse managers should proactively identify and address the causes of missed care by regularly monitoring its impact on patient outcomes. Tools such as the RONCOC can be employed to pinpoint areas where care is delayed or missed, enabling targeted interventions. By adopting these strategies, nurse managers can create a supportive environment that enhances teamwork, reduces missed care, and improves overall care quality.

#### Implications for nursing research

Future studies should focus on further optimizing nursing care quality and patient outcomes by exploring several key areas. Research on the long-term sustainability and scalability of the Big 5 Teamwork Awareness Sessions across diverse healthcare settings is recommended. Investigating the impact of teamwork interventions on various patient outcomes, including safety, satisfaction, and overall quality of care, is also important. Comparative studies should be performed to evaluate the effectiveness of different teamwork training models and their impact on the rationing of nursing care and patient-centered care. Identifying and exploring barriers and facilitators to implementing teamwork-training programs in different clinical environments is essential. Additionally, the integration of technology-based solutions, such as simulation training and virtual collaboration tools, should be explored to enhance teamwork and patient care practices.

## Data Availability

Data is provided within the manuscript or supplementary information files.

## Abbreviations

ANOVA: Analysis Of Variance
Big5TWAS: The Big-Five Teamwork Model
CVI: Content Validity Index
F: F test
NTS: Nursing Teamwork Survey
PCC: Patient-Centered Care
RONC: Rationing Of Nursing Care
RONCOC: Rationing Of Nursing Care Observational Checklist
*r*: Pearson coefficient
SD: Standard Deviation
SPSS: Statistical Package for The Social Sciences
t: t test

## Declarations

### Content of the manuscript

We confirm that the content of the manuscript has not been published or submitted for publication elsewhere.
